# A Pilates Exercise Program as a Therapeutic Strategy in Older Adults with Type 2 Diabetes: Effects on Functional Capacity and Blood Glucose

**DOI:** 10.3390/healthcare13091012

**Published:** 2025-04-28

**Authors:** Beatriz Ruiz-Ariza, Fidel Hita-Contreras, Agustín Aibar-Almazán, María Del Carmen Carcelén-Fraile, Yolanda Castellote-Caballero

**Affiliations:** 1 Department of Health Sciences, Faculty of Health Sciences, University of Jaén, 23071 Jaén, Spain; 2 Department of Educational Sciences, Faculty of Social Sciences, University of Atlántico Medio, 35017 Las Palmas de Gran Canaria, Spain; 3 Department of Health Sciences, Faculty of Health Sciences, University of Atlántico Medio, 35017 Las Palmas de Gran Canaria, Spain

**Keywords:** older adults, type 2 diabetes mellitus, Pilates, physical function, blood glucose concentration

## Abstract

**Background/Objectives:** Older adults with type 2 diabetes mellitus (T2DM) often experience impaired physical function and metabolic control. This study aimed to evaluate the effectiveness of a 12-week Pilates-based intervention on blood glucose concentration and physical function in this population. **Methods:** A randomized controlled trial was conducted with 104 older adults (mean age: 70.6 ± 3.15 years; 70.2% women), all diagnosed with T2DM. The participants were assigned to a control group (CG, n = 52) or a Pilates training group (PG, n = 52). The intervention included 24 Pilates sessions over 12 weeks (2 sessions/week, 60 min each). Outcomes were assessed pre- and post-intervention and included capillary blood glucose, handgrip strength, functional mobility (Timed Up and Go), balance (Berg Balance Scale), and flexibility (Chair Sit-and-Reach Test and Back Scratch Test). **Results:** Compared to the control group, the Pilates group showed statistically significant improvements in blood glucose levels (−4.06 mg/dL (*p* < 0.001; d = 0.68)), handgrip strength (+1.76 kg (*p* < 0.001; d = 0.48)), gait speed (*p* < 0.001; d = 0.53), balance (Berg score) (+2.37 points (*p* < 0.001; d = 0.66)), and flexibility (improvements in upper limbs (BST, d = 0.78–0.98) and lower limbs (CSRT, d = 1.07 right; d = 0.63 left)). **Conclusions:** A 12-week Pilates program led to significant improvements in glycemic control, muscular strength, gait speed, balance, and flexibility in older adults with T2DM. These findings support Pilates as a safe, effective, and adaptable non-pharmacological intervention to promote functional and metabolic health in this population.

## 1. Introduction

Population aging is a social phenomenon of great impact [1]. Demographic and epidemiological transitions have increased life expectancy, but this is not always associated with good health, as it leads to an increase in chronic diseases, disability, and dependency [2,3]. Therefore, a comprehensive care model is needed, focused on maintaining active and healthy aging [4]. In 2023, Spain had 9,063,493 people over the age of 65 (20.65% of the population), with projections indicating growth to 30.4% by 2050. Furthermore, life expectancy is 83.77 years, with differences by sex (86.34 in women and 81.11 in men), placing Spain as one of the countries with the highest life expectancy worldwide, with a notable difference between men and women [5]. This aging of the population leads to an increase in the prevalence of chronic diseases, among which diabetes mellitus (DM) stands out [6].

DM is a metabolic disease characterized by hyperglycemia due to insufficient or impaired insulin secretion [7]. Its lack of control can lead to macrovascular and microvascular complications, increasing the risk of diabetic retinopathy, kidney failure, cardiovascular disease, and amputations [8,9]. Since 2000, the prevalence of DM has increased globally, mainly affecting low- and middle-income countries [10,11]. The International Diabetes Federation (IDF) reports that the number of people with diabetes has increased from 108 million in 1980 to 422 million in 2014 [12]. In 2021, 537 million adults suffered from diabetes, with estimates of 643 million in 2030 and 783 million in 2045 [13]. In Europe, 61 million adults are living with diabetes, with a projected increase to 69 million in 2045 [12]. Specifically, in Spain, the prevalence has grown by 42% since 2019, reaching 14.8%, above the European average (9.2%), with a forecast of reaching 9 million affected people in 2025 [14]. Within DM, type 2 diabetes is the most common type and is characterized by insulin resistance and is associated with factors such as obesity, sedentary lifestyle, and advanced age [15,16,17]. Diagnosis requires a basal glucose level above 126 mg/dL or glucose tolerance level > 200 mg/dL, although the glycosylated hemoglobin (HbA1c) ratio (>6.5%) can also be assessed [18,19]. This type of diabetes can cause metabolic, cardiovascular, and musculoskeletal complications, affecting quality of life [7]. Its consequences include neuropathy, nephropathy, retinopathy, cardiovascular disease, and diabetic foot [8]. It also limits physical health in older adults, affecting muscle strength, flexibility, and balance [20,21].

Treatment varies depending on the type of diabetes, with a balanced diet and physical exercise being recommended for type 2 diabetes [22,23,24]. The World Health Organization (WHO) and the IDF recommend promoting exercise as a key strategy in the prevention and control of diabetes [12,13]. The Pilates method was created in the early 20th century by Joseph Pilates, who developed this discipline by combining elements of gymnastics, yoga, and rehabilitation [25]. His approach seeks to strengthen the body and mind through controlled exercises, improving posture, flexibility, and muscular strength. Initially called “Contrology,” its goal was to achieve physical and mental balance through precise control of movement [26]. This method is based on six fundamental principles: (i) Concentration: focusing on each movement improves the mind–body connection; (ii) Centralization: the body’s “center” (core) is the axis of movement; (iii) Control: precise execution to avoid injury; (iv) Breathing: improves oxygenation and performance; (v) Precision: structured movements to optimize exercise; and (vi) Fluidity: harmonious sequences that promote bodily efficiency [27]. Pilates has demonstrated multiple benefits at the muscular, postural, and mental levels, increasing strength, flexibility, lung capacity, and balance [28]. In older adults with type 2 diabetes, the Pilates method has been shown to be effective in improving glycemic control through mechanisms such as increasing insulin sensitivity, optimizing body composition, and reducing stress [29,30]. However, to comprehensively reflect these physiological effects in a clinical context, it is necessary to evaluate not only metabolic parameters but also functional ones. In this study, five key indicators were selected to capture the overall impact of Pilates in this population. Glucose concentration was included as the main metabolic marker, given its diagnostic and monitoring value in people with type 2 diabetes and its sensitivity to physical exercise interventions [30,31]. Muscle strength, measured by dynamometry, represents an essential component of functional status, particularly affected in older adults with type 2 diabetes due to sarcopenia associated with the disease and aging [32]. Gait speed is a recognized clinical predictor of function, frailty, and fall risk, and responds positively to programs focusing on postural and neuromuscular control such as Pilates [33]. Flexibility and balance are two frequently impaired abilities in this population and have been associated with an increased risk of dependency; they are also core dimensions of bodywork in the Pilates method, which promotes active mobility, core stability, and movement control [34]. Therefore, the selected indicators allow a multidimensional assessment of the effects of Pilates on aspects relevant to both function and metabolic management of type 2 diabetes in older adults.

In previous research conducted by our group, the Pilates method has been shown to be effective in improving muscle strength, postural balance, musculoskeletal pain, sleep quality, and quality of life in healthy older adults [35,36,37,38,39]. These interventions were carried out with protocols similar to those used in the present study, which allowed us to validate their applicability, safety, and progression in this age group. Furthermore, a recent systematic review has highlighted the potential of the Pilates method to improve various physical and psychological parameters in older adults, such as muscle strength, balance, and functionality [40]. However, this review also emphasized the need for more well-designed clinical trials in specific clinical populations, such as those with chronic diseases, to more firmly establish its effectiveness and generalizability. Based on this background, it was deemed pertinent to evaluate the efficacy of the Pilates method in a population with specific clinical conditions, such as older adults with type 2 diabetes mellitus, thus expanding the available evidence on its potential benefits for both functional and metabolic parameters.

## 2. Materials and Methods

### 2.1. Study Design

The design of this research was based on a randomized controlled clinical trial. Before beginning the intervention phase, all participants were informed about the study through a detailed document and gave their written informed consent. Furthermore, it is important to highlight that this study was approved by the Ethics Committee of the University of Jaén under reference FEB.23/3.TES. All phases of this clinical trial were carried out in accordance with the ethical guidelines established in the World Medical Association’s Code of Ethics for the Conduct of Research Involving Human Subjects, known as the Declaration of Helsinki. Furthermore, it is relevant to mention that this clinical trial was registered in the online database Clinicaltrials.gov and assigned the registration number NCT05711602.

### 2.2. Participants

This study included older adults aged 65 years or older who met the following **pre-established inclusion criteria**: (i) medical diagnosis of type 2 diabetes mellitus; (ii) ability to safely perform the exercises included in the Pilates-based program; (iii) not participating in any structured exercise program at the time of recruitment; and (iv) ability to complete all tests and self-administered questionnaires.

The exclusion criteria were as follows: (i) systemic conditions interfering with physical function (e.g., neurodegenerative, musculoskeletal, and severe visual disorders); (ii) vestibular disorders; or (iii) medications affecting balance or coordination (e.g., anxiolytics, antidepressants, and vestibular sedatives).

**Figure 1:**
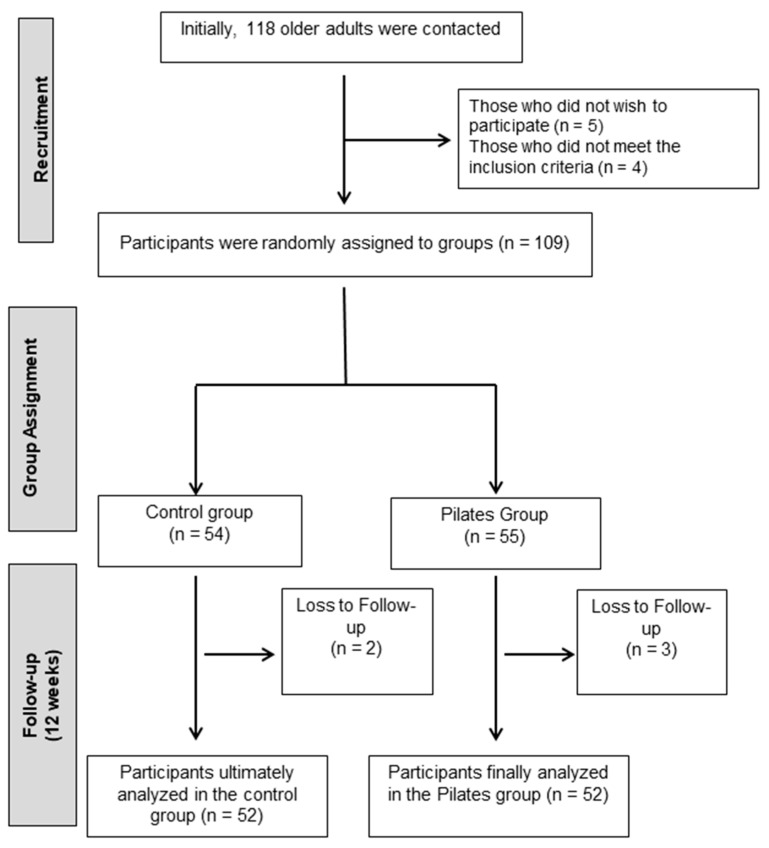
Participant flowchart.

A total of 118 older adults from Jaén were contacted. All were assessed based on the above criteria. Of these, 5 declined to participate and 4 did not meet the inclusion criteria. The remaining 109 individuals were enrolled in the study and randomly assigned to the intervention or control groups (Figure 1).

### 2.3. Randomization

To ensure the quality of the recruitment process and reduce the risk of bias, strict inclusion and exclusion criteria were established. Furthermore, randomization was carried out using opaque envelopes and random numbers generated by an external researcher, without contact with the participants or knowledge of the assigned group. Furthermore, the pre- and post-intervention assessments were conducted by a researcher blinded to group assignment. These strategies helped minimize potential selection and measurement biases. However, given that the sample was recruited in a single city, it is acknowledged that the generalizability of the results may be limited to similar settings. Ultimately, 55 participants were assigned to the Pilates group (EG) and 54 to the control group (CG). Participants assigned to the control group were instructed to maintain their usual daily routines and refrain from participating in any exercise programs. Recommendations aimed at promoting physical activity were provided.

### 2.4. Sample Size Calculation

To calculate the sample size, the statistical program Ene 3.0 was used, based on the data published by Liao et al. [41], for the variable muscular strength, and assuming that the mean of the reference group is 40.89 units, the mean of the experimental group is 44.88 units, and the standard deviation of both groups is 4.34 units, it is necessary to include 20 experimental units in the reference group and 20 units in the experimental group. Considering that the expected dropout rate is 21.70%, it would be necessary to recruit a minimum of 26 experimental units in the reference group and 26 units in the experimental group, totaling 52 experimental units in the study.

### 2.5. Outcomes

All data and variables for this study were gathered by an independent researcher who was unaware of the group assignments and the implementation of the intervention. Data collection took place both before the groups were assigned and after the intervention period was completed. Information on sociodemographic and clinical characteristics was obtained, including age, weight (measured with a high-precision Tefal digital scale ranging from 100 g to 130 kg), height (measured using an Asimed T201-T4 stadiometer), marital status (categorized as married, single, separated/divorced, or widowed), employment status (employed, unemployed, or retired), and educational attainment (no formal education, primary, secondary, or university level). Additional data included the number of years living with the disease, waist circumference (measured at the narrowest point of the abdomen, typically near the navel), hip circumference (taken at the widest part of the hips and buttocks), and waist-to-hip ratio (calculated by dividing the waist measurement by the hip measurement). Body mass index (BMI) was determined as weight in kilograms divided by height in meters squared (kg/m^2^).

#### 2.5.1. Blood Glucose Concentration

Capillary blood glucose was measured using a glucometer, the Accu-Chek^®^ Instant (Roche Diagnostics GmbH, Mannheim, Germany), with standardized test strips and lancets. The measurement was conducted in the morning, approximately 2 h after breakfast, under postprandial conditions. This procedure was applied uniformly across all participants to ensure consistency in data collection. Reference values for postprandial glucose were considered (<180 mg/dL) according to ADA guidelines. This approach reflects routine practice in the Spanish primary care context [42].

#### 2.5.2. Muscle Strength

To measure muscle strength, handgrip strength was assessed using a handheld dynamometer (TKK 5001, Grip-A; Takei, Tokyo, Japan) with a 4.5 cm grip. Participants were asked to perform three attempts at maximal grip strength with their left hand and then with their right, resting for 30 s between each measurement. The highest value obtained was recorded as their grip strength, with values below 20 kg considered indicative of low muscle strength [43].

#### 2.5.3. Physical Function

Physical function was assessed using gait speed with the Timed Up and Go (TUG) test [35]. In this test, participants were required to rise from a seated position, walk three meters, turn around, and sit down again. The time recorded in the TUG test was used to calculate gait speed using the formula [6/(TUG time)]*1.62. The standard threshold of ≤0.8 m/s was adapted to classify walking as slow [44].

#### 2.5.4. Flexibility

Functional flexibility was evaluated using the Back Scratch Test (BST) [45] to assess upper-body flexibility and the Chair Sit-and-Reach Test (CSRT) [46] to evaluate lower-body flexibility. The BST measured shoulder flexibility and was performed in a standing position. Participants reached one hand over the shoulder and down the back, while the opposite hand reached behind the lower back and up toward the other hand. The test was then repeated with the arms reversed. The distance between the tips of the middle fingers was recorded: a “zero” score was assigned if the fingers just touched; a negative value (in cm) was assigned if they did not meet; and a positive value was assigned if they overlapped.

For lower-limb flexibility, the CSRT focused on hamstring flexibility. Participants sat on a chair positioned against a wall for support and reached toward the toes of one leg at a time, keeping the other foot flat on the floor. If the fingers merely touched the toes, the score was zero; if they could not reach the toes, the distance was recorded as a negative value (cm); and if they extended beyond the toes, a positive value was noted.

#### 2.5.5. Balance

Participants’ overall balance was assessed using the Berg Balance Scale (BBS) [47], a clinical test for evaluating static and dynamic balance. This survey contained 14 items answered on a five-point Likert scale on balance-related activities such as sitting and standing without support, with 4 indicating the best performance and 0 demonstrating the worst performance. The total score for the scale can range from 0 to 56 and is obtained by summing the 14 items. Points are based on the time each position is held, the distance the upper limb is able to reach in front of the body, and the time required to complete each task [48].

### 2.6. Intervention

The intervention consisted of 24 structured Pilates sessions over 12 weeks (2 sessions/week, 60 min/session), taught by a certified instructor (with a minimum of 450 h of training) specializing in older adults and therapeutic Pilates. Each session followed a standardized three-phase structure:

Phase 1: Warm-Up (10 min). Gentle breathing (e.g., lateral thoracic breathing), global joint mobilization (cervical circles, shoulder rolls, and hip rotations), spine articulation, and posture activation (neutral spine awareness in standing or seated position).

Phase 2: Core Training (35 min). Exercises were selected and progressed according to safety, neuromuscular control, and functional demand. Weekly progression was as follows (Table 1).

Exercises were modified based on individual ability and progressed by increasing repetitions, changing the base of support, or using implements for resistance.

Phase 3: Cool-Down (15 min). This included global static stretching (hamstrings, hip flexors, and pectorals), diaphragmatic breathing, and body awareness exercises in supine or seated position.

Load was increased when the participant was able to complete the full range of movement in a stable and coordinated manner without compensatory patterns or fatigue signs. Progression included increasing repetitions (from 6 to 12), adding resistance via elastic bands or circles, reducing the support base (e.g., bilateral to unilateral stance), and adding multiplanar movement. Attendance was recorded each session. Exercises were supervised at all times, with continuous feedback and verbal/manual corrections. Participants were instructed to maintain their usual medication and lifestyle during the study.

Throughout the 12-week Pilates program, participant adherence was actively monitored. The overall attendance rate in the experimental group was 87.3%, with the majority of participants attending at least 20 of the 24 sessions. To encourage continued participation and reduce dropout risk, the research team implemented several engagement strategies, including (1) regular phone reminders 24 h prior to each session, (2) motivational messages and check-ins by the supervising instructors, and (3) creating a supportive group environment during sessions to promote social connection. Instructors maintained detailed attendance logs and provided individualized attention to participants expressing difficulties. All sessions were supervised by certified professionals, ensuring safety and fostering a sense of commitment among the participants. These measures contributed to the high adherence rate and the successful completion of the intervention by all participants in the experimental group.

**Figure 2:**
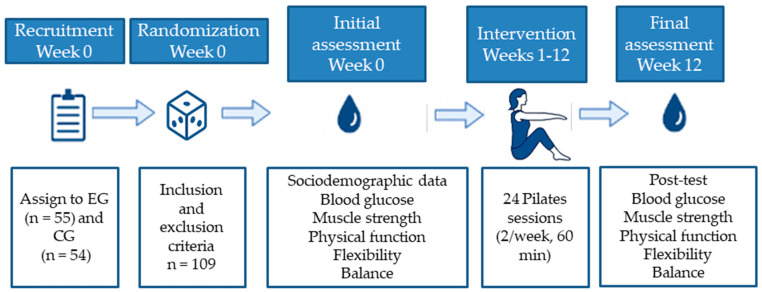
Experimental flowchart of the study design.

A detailed schematic representation of the experimental design is provided below (Figure 2). This flowchart illustrates the key stages of the study, including participant recruitment, group allocation, intervention duration, session structure, and timing of outcome assessments. The aim of this visual summary is to facilitate understanding and ensure the replicability of the protocol.

### 2.7. Statistical Analysis

The statistical analysis was conducted using SPSS software, version 20.0, for Windows (SPSS Inc., Chicago, IL, USA). Statistical significance was established at a threshold of *p* < 0.05. Continuous variables were expressed as means and standard deviations, while categorical variables were summarized as frequencies and percentages. To assess the normality of data distribution, the Kolmogorov–Smirnov test was applied. Prior to the intervention, potential differences between the two study groups were analyzed using the Student’s t-test for continuous variables and the chi-square test for categorical variables. A mixed-design analysis of variance (ANOVA) was used to evaluate possible differences over time and between groups, with the group (control vs. experimental) as the between-subjects factor and the time points (pre- and post-intervention) as the within-subjects factor. The dependent variables analyzed included blood glucose levels, muscle strength (measured via hand-held dynamometry), physical function (Timed Up and Go test), flexibility (assessed by BST and CSRT), and balance (measured with the Berg Balance Scale). Each dependent variable was analyzed separately, and interactions between group and time were explored. To estimate the magnitude of observed effects, Cohen’s d was calculated, considering values < 0.2 as negligible, between 0.2 and 0.49 as small, between 0.5 and 0.79 as moderate, and ≥0.8 as large effect sizes [49].

## 3. Results

This study included 29.81% men and 70.19% women. The mean age of participants was 69.70 ± 6.44 years. Most were retired (57.26%), married (59.83%), and had completed primary education (64.10%) (Table 2). Regarding the group comparison, there were no significant differences in sociodemographic characteristics.

**Figure 3:**
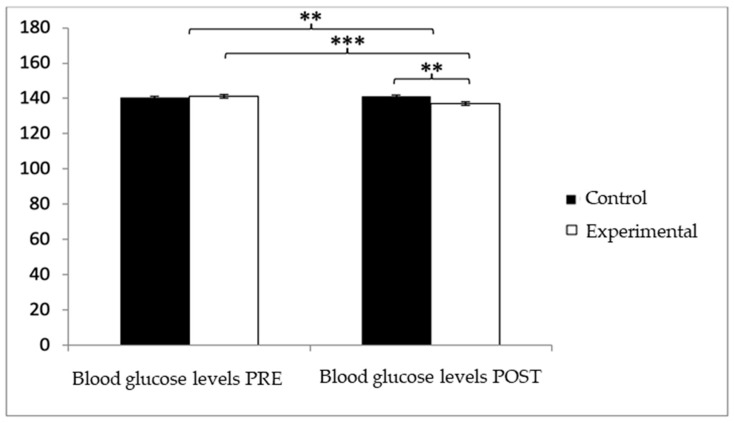
Inter- and intragroup comparisons regarding blood glucose concentration.** *p* < 0.01; *** *p* < 0.001.

With respect to blood glucose levels, prior to the intervention, participants in the experimental group (EG) exhibited slightly higher values (140.05 ± 7.28) compared to those in the control group (CG) (139.47 ± 6.30). However, after the intervention, glucose levels were higher in the CG (140.52 ± 6.71) than in the EG (135.99 ± 6.52). The analysis revealed a significant Group × Time interaction: F(1,102) = 39.377, *p* < 0.001, η^2^ = 0.279, and a significant effect of Time: F(1,102) = 13.556, *p* = 0.001, η^2^ = 0.117, while no significant differences were found for the Group factor alone: F(1,102) = 2.459, *p* = 0.120, η^2^ = 0.024 (Figure 3). Further analysis of the interaction indicated statistically significant differences between both groups in the post-intervention assessment, t(102) = 3.496, *p* = 0.001, with a large effect size (d = 0.68). Additionally, within the experimental group, significant differences were observed between pre- and post-intervention measurements, t(51) = 6.498, *p* < 0.001, showing a medium effect size (d = 0.59).

**Figure 4:**
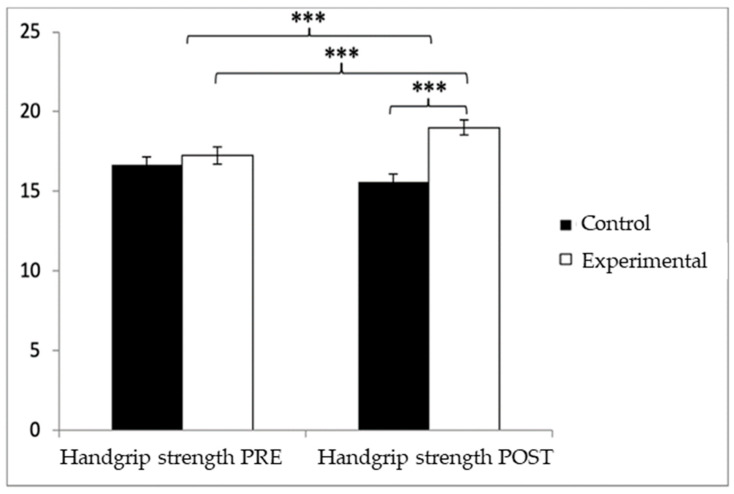
Inter- and intragroup comparisons of muscle strength. *** *p* < 0.001.

Regarding handgrip strength, participants in the EG reported higher values (17.25 ± 3.83) than those in the CG (16.52 ± 3.73) before the start of the intervention, as well as in the post-measure (19.01 ± 3.49 vs. 16.06 ± 3.83). Significant differences appeared in Group × Time: F(1.102) = 45.432, *p* < 0.001, η^2^ = 0.308; Group: F(1.102) = 6.728, *p* = 0.011, η^2^ = 0.062; and Time: F(1.102) = 15.342, *p* < 0.001, η^2^ = 0.131 (Figure 4). The exhaustive analysis of the interaction demonstrates the existence of statistically significant differences between both groups in the post-intervention measure, t (102) = −4.115, *p* < 0.001, with an insignificant effect size (d = 0.19). In addition, the existence of statistically significant differences between the pre- and post-measures was observed in the group that received the Pilates treatment/training, t (51) = −9.457, *p* < 0.001, with a small effect size (d = 0.48).

**Figure 5:**
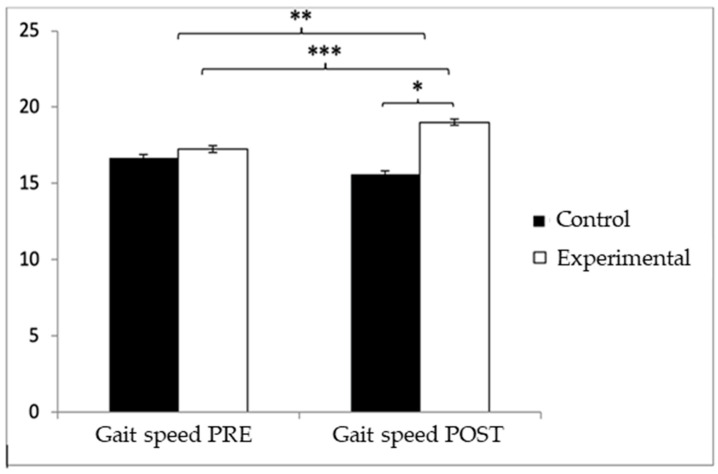
Inter- and intragroup comparisons regarding gait speed. * *p* < 0.05; ** *p* < 0.01; *** *p* < 0.001.

Regarding gait speed, participants in the EG reported higher values (9.72 ± 1.55) than those in the CG (9.49 ± 1.70) before the start of the intervention, but, on the contrary, after the intervention, the CG (9.67 ± 1.57) obtained higher values than the EG (8.98 ± 1.20). Significant differences appeared in Group × Time: F(1.102) = 26.513, *p* < 0.001, η^2^ = 0.206, and Time: F(1.102) = 10.024, *p* = 0.002, η^2^ = 0.089, but not in Group: F(1.102) = 0.64, *p* = 0.4261, η^2^ = 0.006 (Figure 5). The exhaustive analysis of the interaction demonstrates the existence of statistically significant differences between both groups in the post-intervention measure, t (102) = 2.498, *p* = 0.014, with a small effect size (d = 0.49). In addition, the existence of statistically significant differences between the pre- and post-measures was observed in the group that received the Pilates treatment/training, t (51) = 4.934, *p* < 0.001, with a medium effect size (d = 0.53).

**Figure 6:**
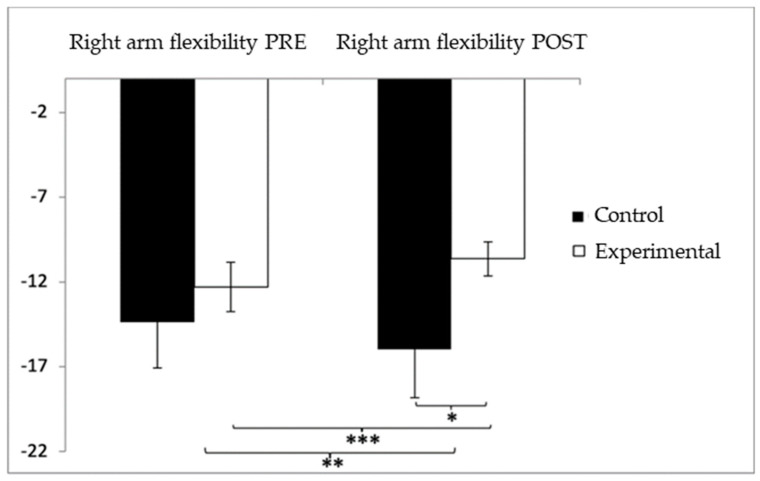
Inter- and intragroup comparisons regarding right-arm flexibility. * *p* < 0.05; ** *p* < 0.01; *** *p* < 0.001.

In relation to upper-limb flexibility (right arm), participants in the experimental group (EG) initially presented lower performance (−12.29 ± 10.45) compared to the control group (CG), who showed values of −8.52 ± 10.10 before the intervention. However, following the intervention, the CG reported higher (worse) scores (−9.54 ± 11.91) than the EG (−5.67 ± 6.46). The analysis identified a significant interaction effect between Group and Time: F(1,102) = 17.181, *p* < 0.001, η^2^ = 0.144, as well as a significant effect of Time: F(1,102) = 9.231, *p* = 0.003, η^2^ = 0.083. No significant effect was found for Group alone: F(1,102) = 0.001, *p* = 0.978, η^2^ = 0.000 (Figure 6). A detailed analysis of the interaction revealed statistically significant differences between the groups in the post-intervention evaluation, t(115) = 2.057, *p* = 0.042, with a small effect size (d = 0.40). Moreover, within the experimental group, significant improvements were observed between pre- and post-intervention measures, t(51) = −7.027, *p* < 0.001, with a medium effect size (d = 0.78).

**Figure 7:**
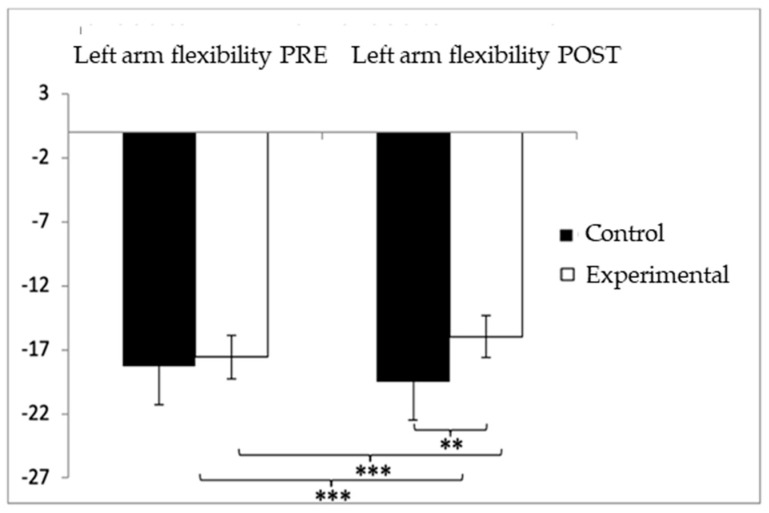
Inter- and intragroup comparisons regarding left-arm flexibility. ** *p* < 0.01; *** *p* < 0.001.

For the left-arm flexibility test, participants in the control group (CG) showed higher (worse) values (−18.35 ± 21.50) compared to those in the experimental group (EG) (−17.33 ± 12.26) prior to the intervention. This pattern persisted after the intervention, with the CG continuing to report higher values (−18.40 ± 21.33) compared to the EG (−7.56 ± 6.93). The analysis revealed significant effects for Group × Time interaction: F(1,102) = 33.983, *p* < 0.001, η^2^ = 0.250; for Time: F(1,102) = 33.190, *p* < 0.001, η^2^ = 0.246; and for Group: F(1,102) = 33.973, *p* < 0.001, η^2^ = 0.250 (Figure 7). A detailed examination of the interaction indicated statistically significant differences between both groups in the post-intervention assessment, t(115) = −3.487, *p* = 0.001, with a medium effect size (d = 0.68). Furthermore, within the experimental group, significant improvements were found when comparing pre- and post-intervention results, t(51) = −6.103, *p* < 0.001, with a large effect size (d = 0.98).

**Figure 8:**
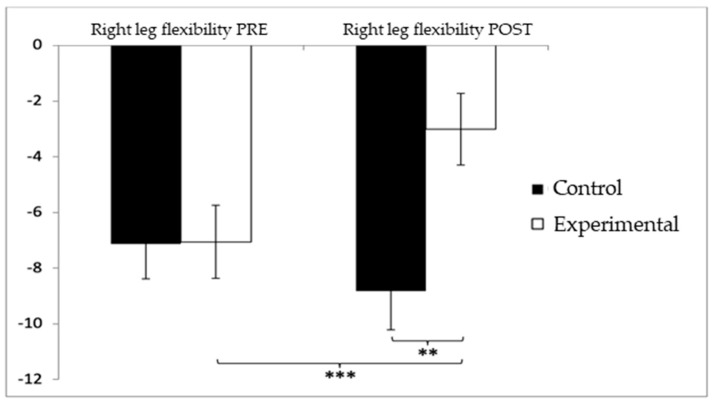
Inter- and intragroup comparisons regarding right-leg flexibility. ** *p* < 0.01; *** *p* < 0.001.

Regarding lower-limb flexibility in the right leg, participants in the control group (CG) initially showed slightly higher (worse) values (−7.13 ± 9.03) compared to the experimental group (EG) (−7.06 ± 9.49) before the intervention. After the intervention, this difference persisted, with the CG reporting higher negative values (−8.83 ± 10.03) compared to the EG (−3.02 ± 9.28). The analysis revealed significant differences for the Group × Time interaction: F(1,102) = 12.312, *p* = 0.001, η^2^ = 0.108, and for the Group factor: F(1,102) = 12.311, *p* = 0.001, η^2^ = 0.108. However, no significant differences were found for the Time factor alone: F(1,102) = 2.067, *p* = 0.154, η^2^ = 0.020 (Figure 8). Further analysis of the interaction confirmed statistically significant differences between both groups in the post-intervention evaluation, t(102) = −3.065, *p* = 0.003, with a large effect size (d = 1.23). Additionally, significant improvements were observed within the experimental group between pre- and post-intervention assessments, t(51) = −3.851, *p* < 0.001, also showing a large effect size (d = 1.07).

**Figure 9:**
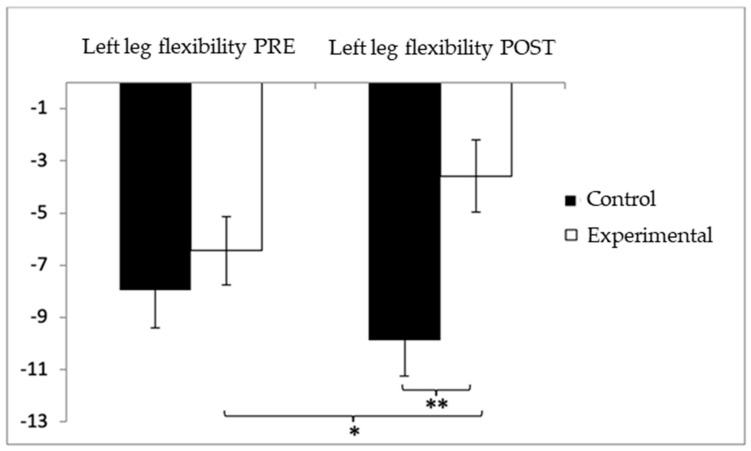
Inter- and intragroup comparisons regarding left-leg flexibility. * *p* < 0.05; ** *p* < 0.01.

Similarly, for lower-limb flexibility in the left leg, participants in the control group (CG) showed higher (worse) values (−7.96 ± 10.31) compared to the experimental group (EG) (−6.44 ± 9.41) before the intervention. This trend continued in the post-intervention assessment, with the CG reporting more negative values (−9.87 ± 9.95) than the EG (−3.58 ± 9.92). Statistical analysis revealed significant differences in the Group × Time interaction: F(1,102) = 8.285, *p* = 0.005, η^2^ = 0.075, and in the Group factor: F(1,102) = 4.941, *p* = 0.028, η^2^ = 0.046. However, no significant differences were detected for the Time factor alone: F(1,102) = 0.346, *p* = 0.563, η^2^ = 0.003 (Figure 9). Further examination of the interaction indicated statistically significant differences between both groups in the post-intervention evaluation, t(102) = −3.227, *p* = 0.002, with a medium effect size (d = 0.63). Additionally, within the experimental group, significant improvements were observed when comparing pre- and post-intervention results, t(51) = −2.521, *p* = 0.015, with a medium effect size (d = 0.30).

**Figure 10:**
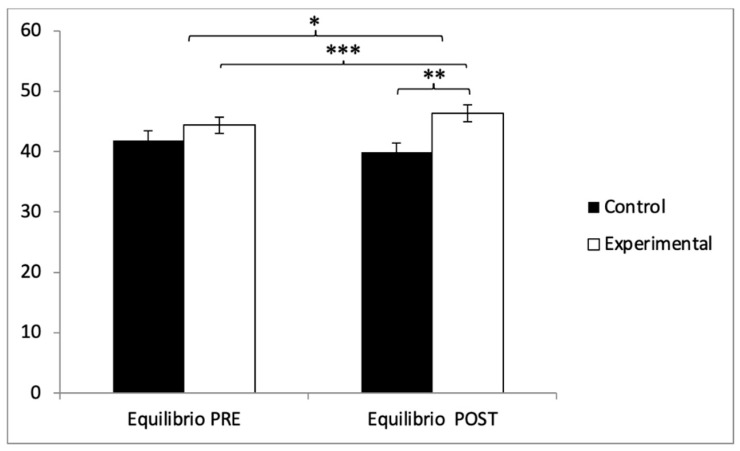
Inter- and intragroup comparisons regarding balance. * *p* < 0.05; ** *p* < 0.01; *** *p* < 0.001.

With respect to balance, participants in the experimental group (EG) exhibited higher scores (44.38 ± 9.81) compared to those in the control group (CG) (41.85 ± 11.06) prior to the intervention. This pattern remained in the post-intervention evaluation, where the EG reported higher values (46.75 ± 9.93) than the CG (39.85 ± 10.98). Statistical analysis revealed significant differences in the Group × Time interaction: F(1,102) = 242.90, *p* < 0.001, η^2^ = 0.704, and in the Time factor: F(1,102) = 5.322, *p* = 0.023, η^2^ = 0.050. However, no significant differences were found for the Group factor alone: F(1,102) = 1.702, *p* = 0.195, η^2^ = 0.016 (Figure 10). Further analysis of the interaction confirmed statistically significant differences between both groups in the post-intervention assessment, t(102) = −3.363, *p* = 0.001, with a large effect size (d = 0.66). Additionally, within the experimental group, significant improvements were observed when comparing pre- and post-intervention measurements, t(51) = −9.233, *p* < 0.001, with a small effect size (d = 0.24).

## 4. Discussion

The primary purpose of this study was to analyze the effects of Pilates on blood glucose concentration, muscle strength, gait speed, flexibility, and balance in older adults with type 2 diabetes mellitus. The results of the study showed significant improvements in blood glucose concentration and significant differences in muscle strength, gait speed and balance, and flexibility in the group that performed the Pilates exercises.

Diabetes mellitus is a chronic disease that affects millions of people worldwide. It is currently a growing topic, as more studies are focusing on its management and prevention, with numerous advances in research being made [11,50]. It is important to emphasize that, despite the advances, this disease requires careful management and a comprehensive approach that involves not only pharmacological treatment but also changes in daily habits. Ongoing medical care, health education, and community support are fundamental pillars for the successful management of this disease [51].

As reflected in Table 3, the most recent systematic reviews agree in pointing out the positive impact of different exercise modalities—aerobic, resistance, combined, and even mind–body—on metabolic, inflammatory, functional, and psychological variables in older adults with type 2 diabetes mellitus. However, many of these interventions have significant methodological limitations, such as protocol heterogeneity, limited duration, and a low quality of evidence. In this context, the present study represents a novel contribution, applying a standardized Pilates program tailored to older adults with type 2 diabetes and evaluating not only functional parameters but also its potential influence on glycemic control. Thus, our work not only addresses the gaps identified in the previous literature but also contributes to expanding the range of safe and potentially effective non-pharmacological interventions for the comprehensive management of this chronic disease in aging.

Maintaining optimal blood glucose levels is essential for the management of diabetes mellitus and for overall metabolic health control. To achieve blood glucose control, it is essential to adopt a comprehensive approach that addresses healthy lifestyle habits, regular glucose monitoring, patient involvement, and collaboration with various healthcare professionals [57]. Over the years, physical exercise has been shown to have a positive effect on the population, highlighting improvements in the quality of life and physical and mental health of older adults during aging. Thus, it constitutes not only a lifestyle but also a complementary therapy for many diseases [58,59]. In the present study, at the beginning of the intervention, the experimental group (EG) reported higher values than the control group (CG), unlike the post-intervention measurements, where the control group obtained higher values. Therefore, significant differences were found between the two groups after the intervention with a large effect size (d = 0.68). Similarly, Melo et al. [60], after their study in which they carried out a Pilates program for 12 weeks, showed significant improvements in glycemia, although in this case the sample was only composed of women between 65 and 67 years old who suffered from T2DM, unlike our study, which also included men with said pathology. Other studies have confirmed the beneficial effects on glucose concentration, but carrying out another type of intervention such as the study carried out by Parra et al. [61], who demonstrated that physical exercise, supervised by professionals, can be of great help in improving the different metabolic parameters in older adults suffering from type 2 diabetes mellitus, such as glycemic concentration values, blood pressure levels, and cholesterol, and reduce cardiovascular risk in adults over 65 years of age. Likewise, Nikkhah Ravari et al. [62] concluded that after completing a 12-week mindfulness program, improvements in glycemic control were obtained in patients with type 2 diabetes mellitus.

Functional capacity in older adults is a widely studied topic in today’s society, as it is a key aspect of quality of life and autonomy during the aging process [63]. Both T2DM and aging itself can affect mobility, vision, and even coordination in these individuals, causing difficulty in performing basic activities of daily living (dressing, grooming, eating, moving, etc.) [64]. To ensure that the functionality of these older adults is not diminished, it is important to maintain and even increase certain parameters, such as muscle strength, gait speed, flexibility, and balance. It is important for healthcare professionals to assess and address this issue in a multidisciplinary manner and provide programs that promote adapted and supervised physical activity with the aim of increasing autonomy in this population [65]. Muscle strength becomes a determining factor in maintaining functional capacity and, therefore, the independence and autonomy of older adults. Aging itself leads to the loss of muscle mass and strength, a process known as sarcopenia [66]. This parameter, however, can be prevented and even improved with sustained physical activity and a healthy diet. Preventing the loss of muscle strength also contributes to good bone and joint health. A comprehensive approach that addresses nutrition, adapted physical activity, and other lifestyle factors can help improve this aspect in older adults [67]. In our study, participants in the experimental group reported higher muscle strength values both at the beginning of the intervention and in the post-intervention phase. The analysis showed statistically significant differences between the two groups in the post-intervention measure, although the effect size was insignificant (d = 0.19). However, differences were also observed between the pre- and post-measurement measures in the Pilates training group, although this effect was small (d = 0.49). Therefore, this confirms that performing Pilates can significantly increase muscle strength in older people with type 2 diabetes mellitus. Some studies reinforce our results, since they confirm that Pilates training can help improve and increase muscle strength, but no studies have been found that include older people with type 2 diabetes mellitus in their samples. For example, Bullo et al. [68] carried out a systematic review in which they did not include older people with type 2 diabetes mellitus but concluded with significant improvements in muscle strength, thus increasing functional capacity and, therefore, the general quality of life of those who performed Pilates training. This study suggests performing Pilates at least twice a week and, if possible, adding complementary aerobic exercises to improve health at various levels. For example, at the cardiovascular level, aerobic activity reduces blood pressure and improves blood circulation, improves lung capacity, and improves physical endurance, allowing people to perform daily tasks with less fatigue, which is essential for maintaining independence. Similarly, Campos de Oliveira et al. [69], in their 12-week study, observed that Pilates can contribute to elbow flexor muscle strength and upper-limb functionality. However, unlike our study, they only included older women without type 2 diabetes mellitus or any other disease. They concluded that Pilates training is a practice that can effectively contribute to greater independence in performing basic activities of daily living that rely on the upper limbs. On the other hand, Gandolfi et al. [70], after a study in which only older women without chronic diseases participated, found significant improvements in their functionality after an adapted Pilates training program carried out once a week for 20 weeks. They also studied the possible bone remodeling as a result of physical activity, but no improvements were observed in this regard, perhaps due to the short intervention period. In a certain way, Pilates favors functional capacity, and although its practice is interesting due to the variety of benefits it produces in older people, other authors such as Markovic et al. [71] compare traditional Pilates training with resistance and balance training, obtaining greater results with the latter.

Another indicator of functional health is gait speed, which can provide highly relevant information regarding the independence and autonomy of older adults with or without medical conditions. A decrease in gait speed may be associated with greater frailty and, therefore, is associated with a higher risk of adverse health events (e.g., falls). Its assessment helps healthcare professionals anticipate potential associated problems [72]. Some causes of decreased gait speed include loss of muscle strength, decreased lung and heart function, and joint or balance problems, among others. Activities aimed at improving these aspects will be of great help in improving gait speed, along with regular and controlled physical activity [73,74]. Our results reported higher gait speed values in the EG before the start of the intervention, but, conversely, after the intervention, the CG obtained higher values. Significant differences were found between both groups in the post-intervention measurement (d = 0.49), and differences were also observed in the pre- and post-measurements in the Pilates training group, with a medium effect size (d = 0.53). Similarly, Newell et al. [75], in their study, demonstrated the positive effect of a short Pilates program, observing that, after 8 weeks of intervention, gait-related parameters improved significantly and that speed increased in older adults who did not suffer from diabetes mellitus. Therefore, we cannot make a realistic comparison with our results.

Flexibility and balance also play an important role in the functional capacity and autonomy of older adults. Flexibility, for its part, is key to maintaining mobility and preventing falls or fractures, as both muscles and joints are less prone to injury, and it also contributes to proper posture [76]. Balance, for its part, is also important for preventing falls, and, in addition, maintaining it helps older adults maintain independence in performing basic activities of daily living, which contributes to quality of life [77]. Some existing strategies to improve these two parameters include exercises that include stretching of all muscle groups and balance, such as Pilates or yoga [67]. Walking and swimming are also low-impact activities that can help improve these parameters. Incorporating these activities into the routine of older adults is a great step toward encouraging and promoting an active lifestyle and healthy aging. In our study, both upper-limb (right and left) and lower-limb (right and left) flexibility were examined. Regarding the upper limbs, the results showed that both arms improved significantly after the intervention. The CG reported higher flexibility values in both arms after the intervention; the right arm showed significant improvements in both groups post-intervention; and, in addition, differences were observed between the pre- and post-measurements in the group that received Pilates training (d = 0.78). The left arm obtained an even larger effect size than the right (d = 0.98). On the other hand, regarding lower-limb flexibility, the control group again reported higher values in both legs in the post-measurement. The right leg showed statistically significant improvements between both groups in the post-intervention measure, with a large effect size (d = 1.23), as did the pre- and post-measures of the Pilates training group (d = 1.07). The differences in the left leg, although significant, were of medium effect size (d = 0.63 and d = 0.30, respectively). Flexibility has been analyzed by other authors, such as Geremia et al. [78], who concluded that a Pilates program can be very useful in helping the aging stage become more active. This study did not specify whether the participants had diabetes mellitus or not; the researchers only excluded those who had mobility limitations or suffered from neurological or psychiatric problems. They found that performing Pilates exercises three days a week (not necessarily consecutively) for 10 weeks increased flexion, abduction, and lateral rotation of both upper limbs. They also observed improvements in hip flexion, and in the lower limbs, the knee also showed improvements in flexibility in both limbs during flexion. Also, Curi et al. [79] observed statistically significant improvements when analyzing upper- and lower-limb flexibility and therefore revealed improvements in life satisfaction after a 16-week Pilates program. In comparison, our study obtained these results with fewer weeks of intervention, and the sample included both men and women, unlike these authors, who only included women over 60 years of age without known pathologies. Regarding balance, the EG reported higher values both at baseline and at post-measurement. The differences were statistically significant after the exhaustive analysis of the interaction between both groups at post-measurement, with a large effect size (d = 0.66). Furthermore, improvements were observed between the pre- and post-measurement in the group that received Pilates training, although the effect size was small (d = 0.24). Some studies did not show improvements in postural stability, such as that of Gabizon et al. [80], who suggested that Pilates training is not task-specific and, therefore, does not improve balance in older adults. On the other hand, other authors did show improvements; for instance, Mesquita et al. [81], in their study conducted in an older female population, showed that Pilates exercises promoted dynamic balance, while neuromuscular therapy (physical therapy) promoted both dynamic and aesthetic balance. Bullo et al. [68], in their study, also concluded that aesthetic and dynamic balance improved after Pilates training. Evidence suggests that Pilates may improve balance in older adults; however, studies addressing this topic have numerous limitations.

Although this study focused on the short-term effects of a 12-week Pilates-based intervention, the potential for long-term health benefits should not be overlooked. The improvements observed in glycemic control and functional capacity suggest that the continued practice of Pilates may help sustain these benefits over time. However, evidence on the persistence of such outcomes remains limited. Future studies should consider long-term follow-up periods and explore the adherence to and feasibility of home-based or community-integrated Pilates programs. Promoting sustained engagement in physical activity, particularly among older adults with chronic conditions, is essential for maintaining functional independence and metabolic health.

### 4.1. Scalability and Implementation Potential

Given its low cost, adaptability, and minimal equipment requirements, the Pilates-based intervention tested in this study could be feasibly implemented in a variety of real-world settings, such as primary healthcare centers, community senior centers, or fitness institutions. Training qualified instructors and integrating such programs into existing health promotion strategies could enhance accessibility for older adults with type 2 diabetes. Furthermore, group-based formats can foster social support and increase adherence, making Pilates a scalable and sustainable non-pharmacological option for functional and metabolic health management in aging populations.

### 4.2. Limitations

It is necessary to point out some limitations of this study. First, this clinical trial was conducted in a very specific population sample: older adults diagnosed with type 2 diabetes mellitus, excluding all those with any type of systemic disease. Second, only the short-term effects of the intervention were evaluated. Third, the study did not record or control for the use of hypoglycemic medications, which could have influenced the observed improvements in blood glucose levels. However, all participants were advised to maintain their usual pharmacological treatment during the intervention period. Fourth, the study did not include more comprehensive metabolic assessments, such as oral glucose tolerance tests (OGTTs) or insulin tolerance tests (ITTs), which could have provided deeper insight into the physiological mechanisms behind the observed glycemic changes. Fifth, the gender distribution in the study was unbalanced, with a higher proportion of female participants. Although this reflects the real-life population of older adults participating in exercise programs, it may limit the generalizability of the results to male individuals. Sixth, while the exercise protocol followed a progressive structure and was closely supervised, individual internal training load (e.g., perceived exertion and physiological markers) was not formally quantified. This may limit the precision of dose–response interpretation. Future trials should consider including standardized load monitoring to enhance replicability and individual response assessment.

## 5. Conclusions

In summary, this study provides evidence that a structured, supervised 12-week Pilates-based exercise program can lead to improvements in glycemic control, muscular strength, mobility, flexibility, and balance in older adults with type 2 diabetes mellitus. While similar outcomes have been observed with other forms of physical activity, this study contributes to the literature by applying a progressive and replicable Pilates protocol tailored to a population that is often underrepresented in clinical exercise trials. The findings support the integration of Pilates as a complementary, low-impact intervention within diabetes management strategies for older adults, especially in community-based or primary care settings. Further research is warranted to examine the long-term sustainability of these effects and to explore sex-specific and individualized responses.

## Figures and Tables

**Table 1:** Weekly structure and progression of Phase 2: Core Training.

Weeks	Exercise Focus	Examples of Exercises	Implements Used
1–2	Familiarization, chair-based training	Seated spine twist, scapular retraction, seated leg raises, pelvic tilts in chair	None
3–4	Standing balance and posture control	Standing marching, lateral leg lifts, mini-squats with arm elevation, wall roll-downs	Light ball (support/feedback)
5–6	Mat-based foundation exercises	Supine pelvic curl, one-leg stretch, toe taps, arm arcs	Magic circle, soft ball
7–9	Progression in stability and strength	Side-lying leg lifts, seated spine twist with resistance, bridge with ball squeeze, seated rows with band	Elastic band, magic circle, soft ball
10–12	Functional integration and challenge	Supine hundred with leg extension, side-kick series, standing band pushes, mat balance with ball	Bands, circle, ball

**Table 2:** Pre-intervention sociodemographic and clinical characteristics of participants as a whole and by group.

		Total (n = 104)	Experimental (n = 52)	Control (n = 52)	*p*-Value
Age		70.57 ± 3.15	70.67 ± 3.21	70.46 ± 3.12	0.632
Sex	Male	31 (29.81)	15 (48.39)	16 (51.61)	0.672
	Female	73 (70.19)	37 (50.68)	36 (49.32)	
Years with the disease		12.63 ± 7.58	13.10 ± 8.41	12.15 ± 6.70	0.720
Occupational status	Retired	75 (51.00)	39 (52.00)	36 (48.00)	0.586
	Worker	3 (2.00)	2 (66.70)	1 (33.30)	
	Unemployed	26 (17.70)	11 (42.30)	15 (57.70)	
Marital status	Single	14 (9.50)	8 (57.10)	6 (42.90)	0.710
	Married	60 (40.80)	28 (46.70)	32 (53.3)	
	Divorced/separated/widowed	30 (20.40)	16 (53.3)	14 (46.7)	
Educational status	No education	12 (8.20)	9 (75.00)	3 (25.00)	0.090
	Primary education	65 (44.20)	34 (52.30)	31 (47.7)	
	Secondary education	20 (13.60)	6 (30.00)	14 (70.00)	
	University education	7 (4.80)	4 (57.10)	3 (42.90)	
Height		1.63 ± 0.11	1.63 ± 0.11	1.64 ± 0.12	0.355
Weight		70.75 ± 10.81	69.41 ± 10.99	71.08 ± 10.55	0.753
BMI		26.57 ± 3.14	26.14 ± 2.98	26.98 ± 3.27	0.585
Waist circumference		95.04 ± 8.84	94.38 ± 8.80	95.69 ± 8.91	0.900
Hip circumference		107.46 ± 7.14	106.96 ± 7.16	107.96 ± 7.15	0.702
Waist–hip ratio		0.88 ± 0.62	0.88 ± 0.67	0.89 ± 0.56	0.214
Blood glucose concentration		139.76 ± 6.86	140.05 ± 7.43	139. 47 ± 6.30	0.368
Handgrip strength		16.89 ± 3.78	17.25 ± 3.83	16.52 ± 3.73	0.941
Gait speed		9.60 ± 1.63	9.72 ± 1.55	9.49 ± 1.70	0.412
Right-arm flexibility		−10.40 ± 10.39	−12.29 ± 10.45	−8.52 ± 10.10	0.711
Left-arm flexibility		−17.84 ± 17.42	−17.33 ± 12.26	−18.35 ± 21.50	0.932
Right-leg flexibility		−7.10 ± 9.22	−7.06 ± 9.49	−7.13 ± 9.03	0.322
Left-leg flexibility		−7.20 ± 9.85	−6.44 ± 9.41	−7.96 ± 10.31	0.932
Balance		43.12 ± 1.48	44.38 ± 9.81	41.85 ± 11.05	0.282

Quantitative variables are presented as means and standard deviations. Qualitative variables are presented as frequencies and percentages. BMI: body mass index.

**Table 3:** Comparison with conventional interventions in T2DM.

Study	Type of Exercise	Main Findings	Main Limitations
Wibowo et al. [52]	Yoga (postural exercises, breathing, relaxation)	Yoga improved muscle strength and cardiorespiratory fitness (low-quality evidence).	Methodological flaws and small samples in most studies.
Leonel et al. [53]	Aquatic training (water aerobics, resistance movements in water)	Aquatic training improved HbA1c (−0.62%), VO_2_peak, BP, and functional capacity.	Few studies included; more high-quality RCTs needed.
Feng, M, et al. [54]	Resistance exercise training (RET), including various forms, such as weightlifting, resistance-band exercises, and machine-based resistance training	The meta-analysis demonstrated that RET significantly improved glycemic control, evidenced by reductions in HbA1c (mean difference [MD]: −0.51%, *p* < 0.0001) and fasting blood glucose (MD: −1.43 mg/dL, *p* = 0.04). Additionally, RET led to significant reductions in triglycerides (MD: −0.32 mmol/L, *p* = 0.03), total cholesterol (MD: −7.08 mg/dL, *p* = 0.005), and LDL cholesterol (MD: −1.91 mg/dL, *p* = 0.05). RET also increased lean body mass and muscle strength but had no significant effects on body weight, fat mass, blood pressure, or heart rate.	The included studies exhibited heterogeneity in intervention protocols, durations, and intensities. The limited number of high-quality randomized controlled trials (RCTs) and variations in participant characteristics may affect the generalizability of the findings. Further high-quality RCTs are needed to confirm these results and explore the long-term effects of RET in this population.
Valenti et al. [55]	Combined aerobic and resistance training, including activities such as treadmill walking, cycling, and weightlifting exercises	The meta-analysis demonstrated that combined aerobic and resistance training significantly improved glycemic control, evidenced by reductions in HbA1c levels. Additionally, participants experienced improvements in lipid profiles, including reductions in total cholesterol and LDL cholesterol, as well as enhancements in insulin sensitivity.	The included studies exhibited heterogeneity in intervention protocols, durations, and intensities. The limited number of high-quality randomized controlled trials (RCTs) and variations in participant characteristics may affect the generalizability of the findings. Further high-quality RCTs are needed to confirm these results and explore the long-term effects of combined training in postmenopausal women with type 2 diabetes.
Xing et al. [56]	Combined aerobic (e.g., walking, cycling) and resistance training (e.g., weightlifting, resistance bands) interventions	The meta-analysis of 14 randomized controlled trials demonstrated that combined aerobic and resistance exercise interventions significantly reduced inflammatory biomarkers associated with inflammaging in middle-aged and older adults with type 2 diabetes mellitus. Specifically, there were significant reductions in interleukin-6 (IL-6), tumor necrosis factor-alpha (TNF-α), and C-reactive protein (CRP) levels. These findings suggest that such exercise interventions can effectively mitigate chronic low-grade inflammation, potentially improving insulin resistance and metabolic health in this population.	The included studies exhibited considerable heterogeneity in intervention protocols, durations, and intensities. Additionally, the overall risk of bias was high, and the certainty of evidence was low for all biomarker outcomes. The small sample sizes and methodological limitations of the included studies limit the generalizability of the findings. Further high-quality, large-scale randomized controlled trials are needed to confirm these results and explore the long-term effects of combined exercise interventions on inflammaging in middle-aged and older adults with type 2 diabetes mellitus.

BMI, body mass index; BP, blood pressure; CRP, C-reactive protein; ECA, ensayo clínico aleatorizado (Spanish for RCT); HbA1c, glycated hemoglobin; HDL, high-density lipoprotein; IL-6, interleukin-6; LDL, low-density lipoprotein; MD, mean difference; RCT, randomized controlled trial; RET, resistance exercise training; SMD, standardized mean difference; T2DM, type 2 diabetes mellitus; TNF-α, tumor necrosis factor alpha; VO_2_peak/VO_2_ max, peak/maximum oxygen uptake.

## Data Availability

The data shown in this study are available upon request from the corresponding author. The data are not available to the public, since, taking into account the sensitive nature of all the questions asked in this study, all participants were guaranteed that the data obtained would be confidential and would not be shared.

## Abbreviations

The following abbreviations are used in this manuscript:

DM	Diabetes mellitus
IDF	International Diabetes Federation
WHO	World Health Organization
BMI	Body mass index
TUG	Timed Up and Go
CSRT	Chair Sit-and-Reach Test
BST	Back Scratch Test
BBS	Berg Balance Scale
